# Chloral in Pityriasis

**Published:** 1876-05

**Authors:** 


Chloral in Pityriasis.—Dr. Martineau states in a French cotemporary, as the result of a large experience, that chloral is a very efficacious, if not certain means of treatment in this rebellious affection. The solution he used was of the strength of about forty grains to each ounce of water, and this he applied to the scalp each morning, by means of a sponge, using slight friction, and allowing it to dry. If the disease is recent, and the lotion is uninterruptedly used for a month, he predicts a certain cure. In the chronic and more obstinate cases, he recommends the continuance of the application of the solution until the disease disappears, as its daily use produces no inconvenience, whilst it relieves the itching.—*Med. and Surg. Reporter.*

